# Plasma and CSF Neurofilament Light (NfL) as Novel Blood‐Based Biomarkers for Alzheimer's Disease Diagnosis

**DOI:** 10.1002/alz70861_108771

**Published:** 2025-12-23

**Authors:** Ahmed Hussein Mohamed Ismail Kira, Mohamed Khaled Ali Mohamed Ali Zid, Mohamed Hosni Kamel Elkellawi, Arwa Ayman Nagah Ahmed Shahin

**Affiliations:** ^1^ Cairo University Faculty of Medicine, Cairo, 11553 Egypt; ^2^ Cairo University Faculty of Medicine, Cairo, Cairo Egypt

## Abstract

**Background:**

Alzheimer's disease is a progressive neurodegenerative disease characterized by the presence of amyloid β (Aβ) plaques and neurofibrillary tau protein in brain tissue. Neurons start to degenerate due to the depositions presumably causing an immunological response that harms brain tissue. Traditional biomarkers, as amyloid‐β peptide, tau proteins, CSF levels (Aβ, tau, and *p* ‐tau), and neuroimaging techniques have been the Gold Standard in AD diagnosis; However, they are often limited and invasive in monitoring and diagnosis Novel blood‐based biomarkers offer an enhanced opportunity for detecting neurodegeneration in brain diseases due to their noninvasiveness, affordability, reliability, and consistency.

**Method:**

This review focuses on recent literature exploring plasma and CSF Nf‐L in AD diagnosis and progression. Comparative analyses of biomarker efficacy using various cross‐sectional and longitudinal studies found that strong associations between Nf‐L levels, cognitive impairment, imaging changes, and other core biomarkers such as Aβ42, T‐tau, and *p* ‐tau.

**Result:**

Plasma and CSF Nf‐L levels were found to be consistently increased in AD. Importantly, they were consistently elevated the in early symptomatic and preclinical stages. CSF Nf‐L was found to correlate strongly with brain atrophy and cognitive decline, even in individuals who are yet to show significant cognitive impairment. Plasma Nf‐L also showed a significant association with AD progression and general neurodegeneration. Though, plasma Nf‐L lacked AD specificity. Notably, plasma Nf‐L is useful in predicting dementia progression and cognitive decline and offers high complementary value alongside traditional prognostic markers. CSF Nf‐L is more useful to detect AD‐specific pathology, while plasma Nf‐L is better monitoring AD progression and large‐scale screening as it’s accessible.

**Conclusion:**

Plasma and CSF Nf‐L are promising biomarkers for Alzheimer’s disease, with complementary roles: CSF Nf‐L is more AD specific. Plasma Nf‐L is more accessible. Further research is needed to explore their diagnostic specificity, integrate them into AD diagnosis and follow‐up, and study their ability to distinguish AD from other neurodegenerative disorders. Their integration into clinical practice could enable earlier diagnosis, improved tracking of disease progression, and enhanced evaluation of therapeutic response in Alzheimer’s disease.

